# Microbial Diversity in Deep-Subsurface Hot Brines of Northwest Poland: from Community Structure to Isolate Characteristics

**DOI:** 10.1128/AEM.00252-20

**Published:** 2020-05-05

**Authors:** Agnieszka Kalwasińska, Arkadiusz Krawiec, Edyta Deja-Sikora, Marcin Gołębiewski, Przemysław Kosobucki, Maria Swiontek Brzezinska, Maciej Walczak

**Affiliations:** a Department of Environmental Microbiology and Biotechnology, Nicolaus Copernicus University in Toruń, Toruń, Poland; b Department of Geology and Hydrogeology, Nicolaus Copernicus University in Toruń, Toruń, Poland; c Centre for Modern Interdisciplinary Technologies, Nicolaus Copernicus University in Toruń, Toruń, Poland; d Department of Microbiology, Nicolaus Copernicus University in Toruń, Toruń, Poland; e Department of Plant Physiology and Biotechnology, Nicolaus Copernicus University in Toruń, Toruń, Poland; f Department of Food Analysis and Environmental Protection, UTP University of Science and Technology, Bydgoszcz, Poland; Kyoto University

**Keywords:** *Bacillus paralicheniformis*, sulfate-reducing bacteria, sulfur-oxidizing bacteria, geothermal brine, microbial communities

## Abstract

Deep-subsurface hot brines in northwest Poland, extracted through boreholes reaching 1.6 and 2.6 km below the ground surface, were microbiologically investigated using culture-independent and culture-dependent methods. The high-throughput sequencing of 16S rRNA gene amplicons showed a very low diversity of bacterial communities, which were dominated by phyla *Proteobacteria* and *Firmicutes*. Bacterial genera potentially involved in sulfur oxidation and nitrate reduction (*Halothiobacillus* and *Methylobacterium*) prevailed in both waters over the sulfate reducers (“*Candidatus* Desulforudis” and *Desulfotomaculum*). Only one archaeal taxon, affiliated with the order Thermoplasmatales, was detected in analyzed samples. Bacterial isolates obtained from these deep hot brines were closely related to Bacillus paralicheniformis based on the 16S rRNA sequence similarity. However, genomic and physiological analyses made for one of the isolates, Bacillus paralicheniformis strain TS6, revealed the existence of more diverse metabolic pathways than those of its moderate-temperature counterpart. These specific traits may be associated with the ecological adaptations to the extreme habitat, which suggest that some lineages of *B. paralicheniformis* are halothermophilic.

**IMPORTANCE** Deep-subsurface aquifers, buried thousands of meters down the Earth’s crust, belong to the most underexplored microbial habitats. Although a few studies revealed the existence of microbial life at the depths, the knowledge about the microbial life in the deep hydrosphere is still scarce due to the limited access to such environments. Studying the subsurface microbiome provides unique information on microbial diversity, community structure, and geomicrobiological processes occurring under extreme conditions of the deep subsurface. Our study shows that low-diversity microbial assemblages in subsurface hot brines were dominated by the bacteria involved in biogeochemical cycles of sulfur and nitrogen. Based on genomic and physiological analyses, we found that the Bacillus paralicheniformis isolate obtained from the brine under study differed from the mesophilic species in the presence of specific adaptations to harsh environmental conditions. We indicate that some lineages of *B. paralicheniformis* are halothermophilic, which was not previously reported.

## Abstract (precis)

Deep-subsurface aquifers, buried thousands of meters down the Earth’s crust, belong to the most underexplored microbial habitats. Although a few studies revealed the existence of microbial life at the depths, the knowledge about the microbial life in the deep hydrosphere is still scarce due to the limited access to such environments. Studying the subsurface microbiome provides unique information on microbial diversity, community structure, and geomicrobiological processes occurring under extreme conditions of the deep subsurface. Our study shows that low-diversity microbial assemblages in subsurface hot brines were dominated by the bacteria involved in biogeochemical cycles of sulfur and nitrogen. Based on genomic and physiological analyses, we found that the Bacillus paralicheniformis isolate obtained from the brine under study differed from the mesophilic species in the presence of specific adaptations to harsh environmental conditions. We indicate that some lineages of *B. paralicheniformis* are halothermophilic, which was not previously reported.

## INTRODUCTION

Terrestrial thermal waters are waters in which the temperature significantly exceeds the annual mean temperature in a given area. They are usually exploited through boreholes or may emerge at the surface as hot springs. The presence of thermal waters below the ground surface is related to the geothermal gradient of around 25 to 30°C/km (1). The dissolution of evaporates in the water of meteoric origin and reaction between some primary minerals of volcanic rocks and magmatic HCl are the main ways that geothermal brines form.

High temperature, pressure, and salinity and highly reducing (oxygen depleted) and often oligotrophic conditions make the deep-subsurface environment extreme (2, 3). Despite this, the presence of relatively abundant bacterial and archaeal communities, viruses, and fungi was documented in thermal waters (4–7).

Due to the relatively easier access to the surface hydrosphere, including hot springs, the knowledge of microbial communities in these habitats is more extensive (8–11) than that in deep-subsurface ecosystems, located several hundred to several thousand meters below ground surface and therefore reachable only through drilled holes (7, 12, 13). Drilling in the Earth’s crust for the exploitation of geothermal energy and mineral resources enables insight into the microbial life in the deep water-bearing strata, which provides unique information on microorganisms’ durability, diversity, and evolution.

Poland has natural sedimentary and fractured aquifers containing thermal waters of different temperatures, ranging usually from 20° to 80 to 90°C (14). Geothermal water resources in the Polish Lowlands are associated with Mesozoic aquifers located in sand formations of the Lower Cretaceous and Lower Jurassic strata (15) at a depth 1,600 to 4,000 m below ground surface (mbgs). These waters are used for heating as well as for balneotherapeutic and recreational purposes. Their operation in the geothermal heat plants is carried out in a closed system of production and injection wells. The water captured by a production well releases its energy in a heat exchanger, after which it is injected into the aquifer with an injection well. The distances between the production well and the injection well are so large that the cooled brine pumped back into the aquifer does not reduce the temperature of the water being taken (16).

For the present study, the mineral waters were extracted from the lower Jurassic sandstones from a depth of 1,640 mbgs in Pyrzyce and 2,578 mbgs in Stargard located in northwest Poland within the Szczecin Trough.

As deep saline groundwaters of northwest Poland remain well separated from the surficial deposits (17), they are not involved in near-surface water circulation. Self-sustaining autochthonous microbiota, highly interconnected through an exchange of geochemical resources, can be found in such habitats (18). Despite many years of investigations using more and more advanced methods, little is still known about how the particular physicochemical characteristics of a subsurface environment contribute to the observed microbial diversity and community structure.

Our study aimed to reveal the diversity and community composition of microbial assemblages in thermal waters (61 and 87°C) of high salinity (total dissolved solids [TDS], 121 and 140 g/liter) extracted from the aquifer at depths of 1,640 and 2,578 mbgs (meters below ground surface) in Pyrzyce and Stargard, Szczecin, northwest Poland. This was conducted using the high-throughput sequencing of 16S rRNA gene amplicons. As searching for isolates, especially from extreme environments, can be useful for practical reasons, the cultivation-based methods also were used to obtain such microorganisms from deep thermal brines. Then, physiological tests, as well as whole-genome sequencing and comparative analysis, were applied to characterize the selected bacterial isolate for the specific ecological adaptations.

## RESULTS

### Chemical and physical analyses of brine samples.

**FIG 1:**
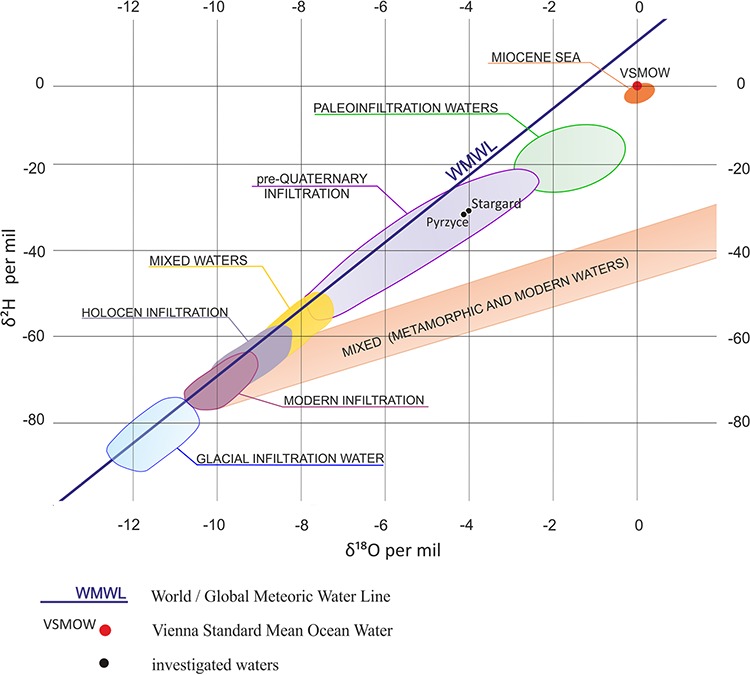
Isotopic composition in Polish waters according to Grabczak and Zuber (88), modified by A. Krawiec.

The waters under study were slightly acidic thermal brines with an outflow temperature of 61°C in Pyrzyce and 85°C in Stargard and high mineralization between 121 and 140 g/liter TDS (Table 1). The major ions were Cl^−^ and Na^+^ (Cl^−^ concentration, 50 and 60 g/liter; Na^+^ content, 42 and 43 g/liter). The concentrations of Ca^2+^ and Mg^2+^ were also high (around 4 g/liter and 2 g/liter, respectively). Both waters were moderately rich in nutrients, except total phosphorous (TP), for which concentrations were below the detection limit, i.e., 0.01 mg/liter. Total organic carbon (TOC) and NH_4_^+^-N concentrations were similar in Pyrzyce and Stargard (30 mg/liter, and 14 to 15 mg/liter, respectively). NO_3_^−^-N and organic nitrogen (ON) contents were significantly higher in Stargard than in Pyrzyce (72 versus 52 mg/liter, and 39 versus 30 mg/liter, respectively). The high total nitrogen (TN)/TP ratios in both waters likely indicated strongly limiting conditions for microbial growth due to P deficiency. The values of δ^18^O and δ^2^H were similar in Pyrzyce and Stargard (ca. 4‰ Vienna standard mean ocean water [VSMOW] and ca. 30‰ VMSOW, respectively) (Table 1). The isotopic composition is close to the world meteoric water line (WMWL), but the values of δ^18^O and δ^2^H indicate heavier isotopic composition (less negative values) than in waters of quaternary infiltration (Fig. 1). Therefore, they can be classified as pre-Pleistocene infiltration waters, which means that they infiltrated more than 2,580,000 years ago. Additionally, the absence of tritium indicates the age is greater than 70 years (19). Due to the higher mineralization, as well as the higher depth of the Stargard’s aquifer, it can be assumed that its water may be older than that in Pyrzyce.

**TABLE 1:** Basic physicochemical and microbiological data of thermal waters from Pyrzyce and Stargard

Parameter^*a*^	Value^*b*^	
	Pyrzyce GT-1	Stargard GT-2
Drilling date	1992	2003
Geochemical data		
Vertical depth (mbgs)	1,637	2,578
Water-bearing stratum	Lower Jurassic, sandstones	Lower Jurassic, sandstones
Temperature (°C)		
At the outlet^*c*^	61	87
In the deposit^*d*^	67	95
δ^18^O (‰ VSMOW)	−4.15	−4.05
δ^2^H (‰ VSMOW)	−30.6	−30.3
Tritium (TU)	0.00	0.00
pH^*e*^	6.2	5.9
TDS (g/liter)	123	129
Cl^−^ (g/liter)	50	60
Na^+^ (g/liter)	42	43
Ca^2+^ (g/liter)	4	4
Mg^2+^ (g/liter)	2	2
SO_4_^2−^ (mg/liter)	119	153
HCO_3_^−^ (mg/liter)	113	95
H_2_S (mg/liter)	+	+
TC (mg/liter)	93	90
TIC (mg/liter)	60	60
TOC (mg/liter)	33	30
NH_4_^+^ (mg/liter)	14	15
NO_3_^−^ (mg/liter)	52	72
ON (mg/liter)	30	39
TP (mg/liter)	<LOD	<LOD
Microbiological data		
TNM (×10^4^ cells/ml)	2.3 ± 1.3	2.1 ± 1.0
TVC (CFU/ml)	3 ± 1	35 ± 4

a VSMOW, Vienna standard mean ocean water; TDS, total dissolved solids; TC, total carbon; TIC, total inorganic carbon; TOC, total organic carbon; ON, organic nitrogen; TP, total phosphorous; TNM, total number of microorganisms; TVC, total viable cells.

b LOD, limit of detection (10 μg/liter); +, trace amount.

c As indicated by the measuring device of the heating system, measured *in situ*.

d See reference 65, measured *in situ*.

e See references 66 and 67, measured *in situ*.

### Abundance of microorganisms in brine samples.

The total numbers of microorganisms, based on direct counting with acridine orange direct cell counting (AODC) technique, were similar in both wells and stayed at the level of 10^4^ cells/ml ([2.3 ± 1.3] × 10^4^ cells/ml in Pyrzyce and [2.1 ± 1.0] × 10^4^ cells/ml in Stargard) (Table 1). The number of thermophilic and heterotrophic microorganisms that grew at 60°C under microaerophilic conditions was lower in Pyrzyce than in Stargard (3 ± 1 CFU/ml and 35 ± 4 CFU/ml, respectively) and constituted from 0.01% to 0.17% of the total number of microorganisms.

### Species richness, diversity, and microbial community structure.

After quality filtering, 27,570 and 30,365 high-quality reads were obtained with bacterial and archaeal 16S rRNA gene primer sets, respectively. Taxonomic assignment of the reads showed that bacterial 16S rRNA genes were amplified with the archaeal primer set. These reads were discarded from the data set and only one archaeal operational taxonomic unit (OTU) remained. Sequences obtained from the negative controls were also removed. The list of genera found in negative controls is reported in Table S1 in the supplemental material at https://www.bio.umk.pl/panel/wp-content/uploads/Supplementary-Material.pdf.

The rarefraction curves presented in Fig. S1 (https://www.bio.umk.pl/panel/wp-content/uploads/Supplementary-Material.pdf) indicate that a high coverage of the samples’ diversity was captured. Bacterial diversity was low. Barely 64 bacterial OTUs were generated for the whole data set. The observed numbers of species were between 10 ± 1 in Pyrzyce and 27 ± 8 in Stargard. The Shannon indices did not differ significantly between brine extracted from Stargard and the water taken from Pyrzyce (1.53 ± 0.1 and 2.5 ± 0.3, respectively).

**FIG 2:**
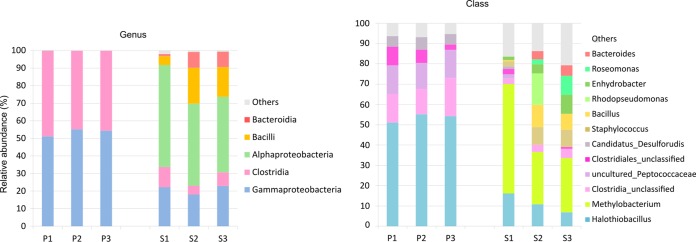
Bacterial community structures in thermal brines from Pyrzyce (P1 to P3) and Stargard (S1 to S3) based on next-generation sequencing reads.

Both thermal waters were dominated by *Proteobacteria* (54% and 70% on average in Pyrzyce and Stargard, respectively) followed by *Firmicutes* (46% and 22%, on average, respectively) (Fig. 2). Sequences assigned to *Bacteroidetes*, *Actinobacteria*, *Acidobacteria*, and *Deferribacteres* were present exclusively in Stargard (6, 0.5, 0.4, and 0.1%, respectively). *Gammaproteobacteria* (54%) followed by *Clostridia* (46%) were the most common in the community structure at the class level in Pyrzyce, while *Alphaproteobacteria* (49%), *Gammaproteobacteria* (21%), *Bacilli* (14%), and *Bacteroidia* (6%) were the most prominent components of the bacterial assembly in Stargard.

Detailed analysis of the bacterial community composition at the genus level showed that *Halothiobacillus* (54%) and two members of unclassified genera within *Clostridia* (28% in total) prevailed in Pyrzyce, while *Methylobacterium* (35%) and *Halothiobacillus* (11%) were the most abundant genera in Stargard (Fig. 2). The potential endospore-forming *Firmicutes* (EFF) constituted an abundant group of microorganisms (in both waters with an average share of 25% in Pyrzyce and 10% in Stargard). These included the following genera: *Bacillus*, “*Candidatus* Desulforudis,” *Clostridium sensu stricto* 10, *Desulfitibacter*, *Desulfosporosinus*, *Desulfotomaculum*, and also one unclassified and one uncultured genus within *Peptococcaceae*. Sequences unclassified at the genus level constituted 39% of the total number of reads in Pyrzyce and 7% in Stargard.

Bacterial communities did not differ significantly as shown by nonparametric multivariate analysis of variance (PERMANOVA; *F *= 12.62, *P* = 0.094). Six bacterial OTUs (9.4% of the total number of OTUs) were shared among samples. The shared OTUs present in both thermal waters were *Halothiobacillus*, “*Candidatus* Desulforudis,” *Desulfotomaculum*, and three unclassified OTUs within *Clostridia* (Table 2).

**TABLE 2:** The shared bacterial OTUs in thermal waters from Pyrzyce and Stargard

OTU	P1	P2	P3	S1	S2	S3	Taxonomy
OTU01	49.2	53.1	51.9	13.7	11.5	5.6	*Proteobacteria*, *Gammaproteobacteria*, *Chromatiales*, *Halothiobacillaceae*, *Halothiobacillus*
OTU11	6.7	1.0	7.0	2.3	3.4	3.7	*Firmicutes*, *Clostridia*, *Clostridia*_unclassified
OTU10	8.8	6.3	2.5	2.2	0.0	0.7	*Firmicutes*, *Clostridia*, *Clostridiales*_unclassified
OTU05	13.6	12.3	13.3	1.8	0.0	0.0	*Firmicutes*, *Clostridia*, *Clostridiales*, *Peptococcaceae*, uncultured_*Peptococcaceae*
OTU07	6.8	11.0	10.3	0.0	0.4	0.0	*Firmicutes*, *Clostridia*, *Clostridia*_incertae_sedis, unknown_family, “*Ca*. Desulforudis”
OTU12	5.1	6.0	4.9	1.0	0.0	0.0	*Firmicutes*, *Clostridia*, *Clostridiales*, *Peptococcaceae*, *Desulfotomaculum*

**FIG 3:**
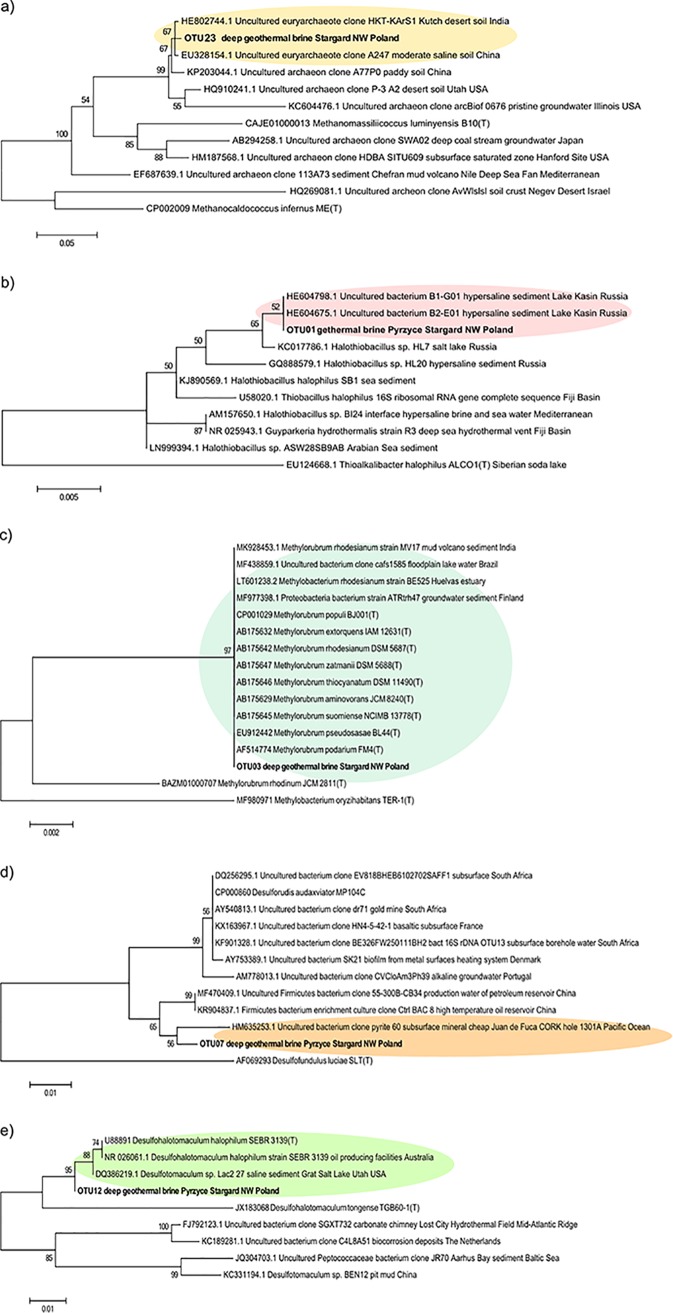
Phylogenetic trees of the most abundant and shared reads retrieved from geothermal brines (Pyrzyce and Stargard, northwest Poland) and other related environmental sequences and type strains based on partial 16S rRNA genes. (a) Unclassified *Thermoplasmatales* OTU23; (b) *Halothiobacillus* OTU01; (c) *Methylobacterium* OTU03; (d) “*Ca*. Desulforudis” OTU07; (e) *Desulfotomaculum* OTU12. The tree was reconstructed using 426 bacterial and 386 archaeal nucleotide positions. GenBank accession numbers, names, and isolation sources of type strains (T)/isolates/clones are indicated.

The only archaeal OTU found in the archaeal data set was unclassified at genus and at family levels. It was assigned to order Thermoplasmatales, class Thermoplasmata, and phylum Euryarchaeota. The most related sequences obtained for this OTU were found in Kutch desert soil, India (Fig. 3).

The most abundant bacterial OTUs (*Methylobacterium* and *Halothiobacillus*) and the most prevailing representatives of sulfate-reducing bacteria (SRB; *Desulfotomaculum* and “*Ca*. Desulforudis”) were related to sequences retrieved from various, mostly saline and high-temperature, environments such as sediments of Lake Kasin, Russia, groundwater sediment in Finland, borehole systems in the Pacific Ocean, and high-temperature oil reservoirs in China (Fig. 3).

### Bacterial isolates.

Bacterial isolates retrieved in this study (see Table S2 at https://www.bio.umk.pl/panel/wp-content/uploads/Supplementary-Material.pdf) belonged to the phylum *Firmicutes* and mostly to the species Bacillus paralicheniformis (nine strains of ten). One strain (TP8) may represent a new species, as the similarity of the 16S rRNA gene to the known type strains was below 95% (20).

### Physiological differences between *Bacillus* strains TS6 and KJ-16.

The study revealed that TS6 can be distinguished from *B. paralicheniformis* KJ-16, which was isolated for the first time from fermented soybean paste (21), based on the higher temperature optimum of 50°C, lack of urease production, the ability to assimilate d-galactose, *myo*-inositol, and d-galacturonic acid, and the susceptibility to nalidixic acid (Table 3).

**TABLE 3:** Phenotypic properties, fatty acid profiles, and indicators of a thermotype calculated for *B. paralicheniformis* strains

Property	Bacillus paralicheniformis strain	
	TS6	KJ-16
Phenotypic properties		
Optimum temperature (°C)	50	37
Optimum pH	7	7–8
Optimum salinity (% NaCl [wt/vol])	3	No data
Urease (Stuart’s urease medium)	−	+
Biology phenotype array		
Succinic acid	+	+
d-Glucuronic acid	−	−
Tween 20	−	−
Tween 40	−	−
Tween 80	−	−
Stachyose	−	−
Glycyl l-glutamic acid	−	−
l-Pyroglutamic acid	−	−
Biolog Gen III plates		
d-Galactose	+	−
*myo*-Inositol	+	−
l-Alanine	+	+
d-Galacturonic acid	+	−
Mucic acid	−	−
Nalidixic acid	+	−
Fatty acid profiles (%)^*a*^		
iso-C_14:0_	0.8	0.6
C_14:0_	0.4	0.6
iso-C_15:0_	41.0	31.5
anteiso-C_15:0_	23.0	37.7
C_16:1_ω7c	0.5	<0.4
iso-C_16:0_	4.0	2.7
C_16:1_ω11c	0.5	0.6
C_16:0_	3.7	5.3
iso-C_17:1_ω10c	1.5	0.4
iso-C_17:0_	15.2	8.9
anteiso-C_17:0_	9.3	11.3
HAI^*b*^	2.0	1.0
a15/i15^*c*^	0.6	1.2
Thermotype	Highly thermotolerant	Mesophilic

a Values are percentages of total fatty acids; only fatty acids making up more than 0.4% of total are shown.

b HAI, heat adaptation index.

c a15/i15, anteiso- to iso-C_15:0_ FA ratio.

The total cellular fatty acid profiles showed large amounts of branched fatty acids (FA) in both strains. Strain TS6 showed higher contents of iso-C_15:0_ (40.7% of the total cellular FA), iso-C_17:0_ (15.2%), and iso-C_16:0_ (4.0%) and smaller amounts of anteiso-C_15:0_ (22.97%), anteiso-C_17:0_ (9.26%), and C_16:0_ (3.7%) than strain KJ-16 (Table 3). Based on the high value of the heat adaptation index (HAI) and low anteiso- to iso-C_15:0_ FA (a15/i15) ratio, strain TS6 can be considered a highly thermotolerant species (22). In contrast, strain KJ-16 had a lower HAI and higher a15/i15 ratio; therefore, it should be assessed as mesophilic. A comparative analysis between the FA profiles for the strain TS6 and those in the database of the TSBA6 (v. 6.10) library of MIDI showed the highest similarity index (0.431) with Bacillus pumilus GC subgroup B.

### *Bacillus* strain TS6 genome sequencing and comparative analysis.

**FIG 4:**
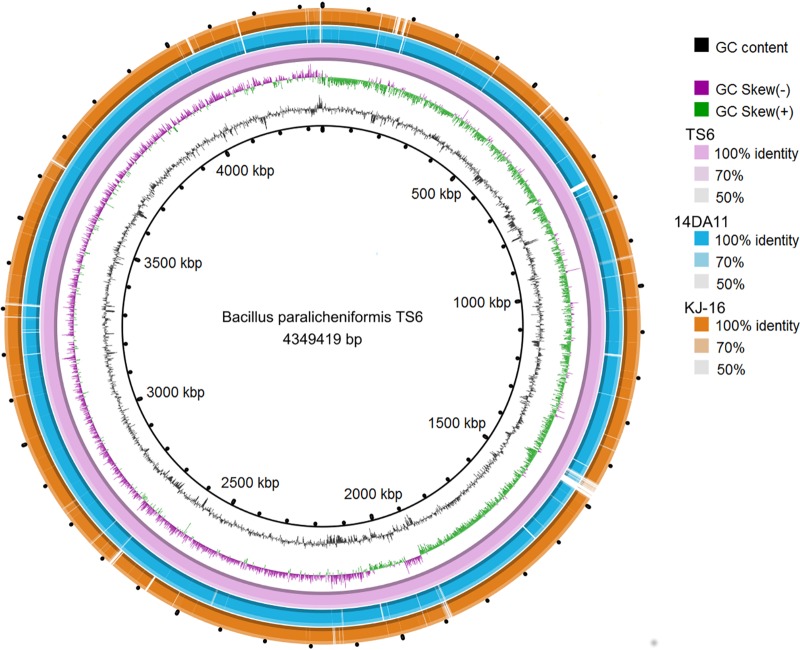
BRIG visualization of the three *B. paralicheniformis* genomes. The two innermost rings show GC content (black) and GC skew (purple/green). The third innermost ring shows TS6 draft genome, followed by the complete reference genome of 14DA11 and the KJ-16 draft genome.

In total, 468,046 reads were obtained from the whole-genome sequencing of the TS6 strain. These were assembled into 31 contigs (>500 bp), giving a consensus length of 4.3 Mbp with 45.9% G+C content at a mean coverage of 38.24 (Fig. 4). Annotation using the NCBI Prokaryotic Genome Annotation Pipeline (PGAP) identified 4,179 coding genes, 116 RNAs, and 164 pseudogenes (see Table S3 at https://www.bio.umk.pl/panel/wp-content/uploads/Supplementary-Material.pdf).

*In silico* DNA-DNA hybridization (DDH) at >70% revealed a similarity value of 91.7% for TS6 versus *B. paralicheniformis* KJ-16 and 95.0% for TS6 versus *B. paralicheniformis* 14DA11, confirming that both strains belonged to the same species; however, the second parameters for DDH at >79% of 67.85% and 72.83%, respectively, may suggest the presence of subspecies within *B. paralicheniformis* (23).

**FIG 5:**
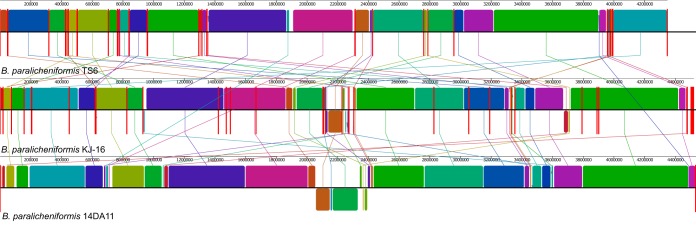
Multiple whole-genome alignment of the three strains of Bacillus paralicheniformis. Homologous blocks in each genome are shown as identically colored regions linked across genomes. Regions that are inverted relative to *B. paralicheniformis* TS6 are shifted below a genome’s center axis.

Comparative genome analysis revealed 70 locally colinear blocks conserved among the three taxa. Significant differences in the arrangement of the homology blocks between the *B. paralicheniformis* TS6 genome, the reference genome, and the genome of its closest relative type strain KJ-16 were discovered. The homology block layout in the 14DA11 genome was quite similar to the *B. paralicheniformis* KJ-16 genome, while the genome of *B. paralicheniformis* TS6 showed a high degree of rearrangements (Fig. 5).

**FIG 6:**
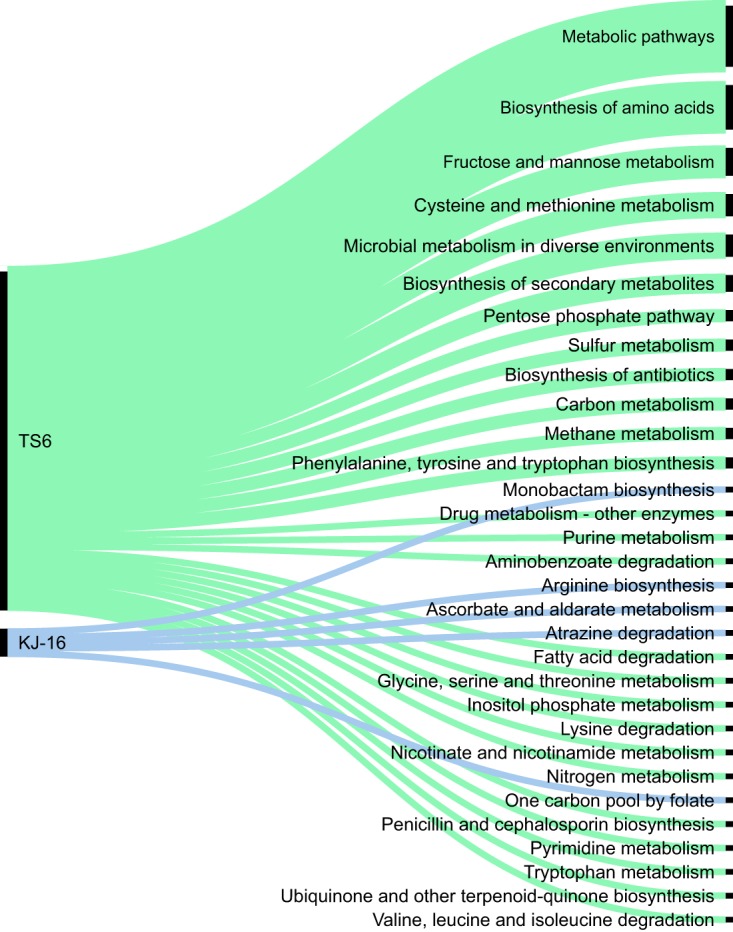
Differences between *B. paralicheniformis* TS6 and *B. paralicheniformis* KJ-16 in the numbers of genes assigned to KEGG metabolism categories.

The *B. paralicheniformis* TS6 genome contained more genes which matched various KEGG pathway categories than *B. paralicheniformis* KJ-16 (Fig. 6). The most numerous groups of these genes were those associated with “metabolic pathways” (11 genes, including *hxlA* and *hxlB*), “biosynthesis of amino acids” (8 genes), “fructose and mannose metabolism” (5 genes), “cysteine and methionine metabolism” (4 genes), “microbial metabolism in diverse environments” (4 genes), and “biosynthesis of secondary metabolites” (3 genes).

On the other hand, a few genes which were present in the genome of *B. paralicheniformis* KJ-16 were not detected in the genome of strain TS6. These were involved in “atrazine degradation,” “biosynthesis of arginine and monobactam,” “ascorbate and aldarate metabolism,” and “one carbon by pool folate.”

## DISCUSSION

Highly mineralized slightly acidic waters from deep geothermal aquifers exploited in Pyrzyce and Stargard present unique environments that are relatively rich in organic carbon and nitrogen but extremely poor in phosphorous. Isotopic studies suggest geological isolation of the lower Jurassic aquifer that does not contain the admixture of contemporary waters (17). The relatively high concentration of NH_4_^+^ and organic carbon may result from anoxic degradation of organic matter and/or interaction between the hydrothermal fluids and sediment containing ammonium-bearing minerals (24, 25).

The high temperature and salinity might have been among the strongest environmental factors that selected specialized microbial communities able to thrive in this polyextreme environment. Under the high temperature and salinity combined with P-deficient conditions, microbial cell densities in the thermal waters of Pyrzyce and Stargard were low (10^4^ cells/ml). Comparable results were obtained by Itävaara et al. (26) and Miettinen at al. (27) at 1,500, 1,430, and 1,350 m below the ground surface. At depths greater than 3 km, Kieft et al. (28) and Borgonie et al. (29) recorded even lower cell densities (10^3^ cells/ml at 3,100 m and 2 to 6 cells/ml at 3,600 m, respectively). A long-lasting physical isolation from biota above the deep aquifer, which in the case of the studied brines may be dated at millions of years old, may serve as a driver toward the emergence of new subpopulations. Under such conditions, microbial communities often exhibit strong regional and local diversification (30). Accordingly, the proportions of unclassified bacterial and also archaeal taxa constituted a significant part of microbial communities in the studied waters.

The relative abundance of the archaeal community, captured with the primer set used, was much smaller than the bacterial one. The residual archaeal sequences detected in thermal brines from Pyrzyce and Stargard, unclassified at the family and genus levels, belonged to Thermoplasmatales. Their cultured representatives were isolated from high-sulfur, natural geothermal habitats like hydrothermal vents. Sequences obtained from this study were closely related to those of environmental clones obtained from the Kutch desert in India, moderately saline soil in China, and a pristine groundwater aquifer in Illinois, USA (31).

Bacterial communities in the studied brines were dominated by *Proteobacteria* and *Firmicutes*, which is in line with observations made by other researchers in geothermal ecosystems (11, 18, 32). Interestingly, the most abundant representatives of *Proteobacteria* were sulfur oxidizers affiliated with *Halothiobacillus* and *Methylobacterium*, which dominated bacterial communities in Pyrzyce and Stargard, respectively.

Representatives of *Halothiobacillus* are obligate chemolithoautotrophs, assimilating CO_2_ via the Calvin-Benson-Bassham cycle at the expense of the oxidation of reduced sulfur compounds (33). Some representatives of the genus *Halothiobacillus*, *H. hydrothermalis* and H. kellyi, were retrieved from marine hydrothermal vent systems (34). The most similar phylotypes related to *Halothiobacillus* from Pyrzyce and Stargard were retrieved by Emmerich et al. (35) from hypersaline sediments of Lake Kasin, Russia. Recently Halothiobacillus hydrothermalis and Halothiobacillus halophilus, showing a more halophilic lifestyle than *Halothiobacillus* sp. *sensu stricto*, were reclassified to a new genus, *Guyparkeria* (33).

The genus *Methylobacterium* consists of methylotrophic bacteria (36) that, in the presence of methylated sulfur compounds such as dimethyl sulfide, are capable of sulfur oxidation (37). Due to the great phenotypic plasticity, members of *Methylobacterium* occupy different habitats, such as drinking water (38), seawater (39), soil (40), and plants (41). Sequences belonging to this genus were also retrieved from subsurface aquifers (42, 43), including hydrothermal environments (44–46). One of the most similar environmental sequences related to the *Methylobacterium* OTU found in Stargard brine belonged to a strain isolated from groundwater sediment in Finland. In 2018, some *Methylobacterium* species were reclassified into a new genus, *Methylorubrum* (47).

*Firmicutes* are considered important members of subsurface microbial communities (7, 13, 48). The high proportion of potentially endospore-forming genera within *Bacilli* and *Clostridia* noted in the saline, deep thermal waters, ranging from 7% of the total bacterial community in Stargard to 39% in Pyrzyce, is consistent with the conclusions made by Filippidou et al. (49). The authors postulated that multiple limiting environmental conditions such as high temperature, low or high pH, high pressure, and exposure to cell-toxic chemical compounds favor the prevalence of EFF. The improved resistance of microorganisms from this group is caused by the combination of sporulation and many additional survival mechanisms, such as chemotaxis, motility, biofilm formation, DNA uptake, and metabolic diversity. It is therefore not surprising that the shared bacterial community of deep and extreme thermal brines of Pyrzyce and Stargard comprised such endospore-producing organisms such as “*Candidatus* Desulforudis” and *Desulfotomaculum*.

“*Candidatus* Desulforudis” and *Desulfotomaculum* are recognized as sulfate-reducing bacteria (SRB) and play important roles in sulfur cycling. Interestingly, the genome of the first organism was obtained from deep-fracture water collected at the depth of 2,800 m in a South African gold mine (50). It was also found as a member of SRB in sulfidic water of Busko-Zdroj at a depth of 600 m (51) and in Fennoscandian fracture fluids at a depth of 180 to 2,260 m, with the highest representation of this genus below 1,820 m (52). Similarly, representatives of the genus *Desulfotomaculum* were commonly found in deep-subsurface environments such as aquifers (53) and hydrothermal vents (54). The most similar phylotypes related to “*Ca*. Desulforudis” and *Desulfotomaculum* from Pyrzyce and Stargard were retrieved from borehole systems in the Pacific Ocean at a site where warm (∼64°C) basement fluids discharge at the seafloor (55) and a high-temperature oil reservoir in China (56), respectively.

Among *Firmicutes*, unclassified *Peptococcaceae* within *Clostridia* presented a significant component of the microbial community in Pyrzyce. Family *Peptococcaceae* comprises anaerobic, mesophilic, or moderately thermophilic organisms capable of utilizing sulfate and thiosulfate (SRB) as well as bacteria using fumarate or ferric iron (Fe^3+^) as electron acceptors (57), thus playing an important ecological role in a subsurface environment not only in sulfur but also in iron cycling.

Our research showed that the thermal brines were inhabited by both the sulfur-oxidizing bacteria (SOB) and sulfate-reducing bacteria (SRB), with the overwhelming share of the former group. According to Schwermer et al. (58), nitrate concentration exceeding 0.8 mM (ca. 49.6 mg/liter) inhibits sulfate reduction and induces sulfide oxidation. Regarding this, the concentrations of nitrates in the water of Pyrzyce and Stargard were high enough (51.8 and 72.2 mg/liter) to decrease SO_4_^2−^ reduction by SRB (e.g., by the representatives of the family *Peptococcaceae* and “*Candidatus* Desulforudis”) and induce NO_3_^−^ reduction coupled with sulfide oxidation by SOB (e.g., by *Methylobacterium* and *Halothiobacillus*). This was further confirmed by a geochemical model proposed by Lau et al. (18), which showed that under the subsurface conditions, the redox couple of sulfur oxidation and nitrate reduction may be energetically more favorable than sulfate reduction.

Although the differences in community compositions were not statistically significant, the bacterial communities of high-temperature brine in Pyrzyce and Stargard showed a relatively low degree of overlap. Moreover, the more saline and higher-temperature water of Stargard was characterized by higher microbial diversity indices than mineral waters of Pyrzyce. Although some studies indicated bacterial diversity reduction with the increase of temperature in geothermal habitats (11, 59), we have observed an opposite trend. It seems possible that in the studied waters, the site-specific factors, i.e., availability of carbon/nitrogen sources as well as electron donors/acceptors, were responsible for differences in bacterial diversity (60).

In this study, the number of heterotrophic culturable bacteria growing at 60°C under microaerophilic conditions was low and did not exceed 10^2^ CFU/ml. The cultivability of the deep-subsurface microbiota was lower than 0.2% of the total number of microorganisms obtained from microscopic analysis. However, these values were from few to several times higher than those usually obtained from great depths (61). Most of the isolates retrieved from the studied brines were assigned to *B. paralicheniformis*. Since it was validly described in 2015, more than fifty genomes of *B. paralicheniformis* retrieved from various samples, e.g., food products, plant and animal tissues, and environmental samples such as Red Sea microbial mat or salty African lake, were sequenced and deposited in the NCBI database.

The whole-genome analysis of strain TS6 (isolated from the Stargard sample) supports its taxonomical assignment to the species *B. paralicheniformis*. However, differences in the genome organizations and physiological traits between TS6 and KJ-16 lead to the conclusion that strain TS6 represents a distinct subspecies within the lineage *paralicheniformis*. In contrast to strain KJ-16, TS6 is a highly thermotolerant organism adapted to high temperature in the deep-subsurface aquifer, which was not previously reported, and may be assigned to a group of bacilli comprising thermophilic species such as Geobacillus stearothermophilus and thermotolerant Bacillus badius (62).

Extensive functional comparisons of strain TS6 to its closest type strain KJ-16 and to the closest reference genome (strain 14DA11), isolated from high-salt fermented Korean soybean, showed that the TS6 genome is enriched in genes involved in the metabolism of biogenic elements (i.e., carbon, nitrogen, and sulfur) as well as simple compounds (i.e., methane, carbohydrates, and amino acids). A good example of an additional metabolic track in the TS6 genome is the ribulose monophosphate (RuMP) pathway. Genes encoding 3-hexulose-6-phosphate synthase (*hxlA*) and 6-phospho-3-hexuloisomerase (*hxlB*) were involved in formaldehyde fixation in many methylotrophic bacteria (e.g., Brevibacillus brevis). They were also important for nonmethylotrophic organisms (e.g., Bacillus subtilis), participating in the detoxification and assimilation of formaldehyde, which is ubiquitous in nature in the form of methylesters of pectin and of the methoxyl group of lignin (63). Both pectin and lignin are components of buried organic matter (64). These and many other additional genes may be related to the increased metabolic potential of a cell that needs to efficiently use all nutrients possibly available in the extremely poor environment or to protect itself against toxins. Additionally, the higher diversity of biosynthesis genes possibly increases the level of cell metabolic fitness that can regulate specific metabolic pathways depending on the environmental conditions.

The present study on microbial communities in deep geothermal brines of northwest Poland demonstrates the low diversity of bacterial assemblages, which is consistent with the previous observations that polyextreme habitats select for specific groups of microorganisms able to survive under harsh conditions. We suspect that the high temperature and salinity, P-deficiency, isolation from the above strata, and site-specific geochemistry were responsible for the remarkable diversity and structure of the bacterial communities, with the predominance of *Halothiobacillus* and *Methylobacterium*. The significant proportions of the putative EFF and bacteria potentially involved in sulfur oxidation coupled with nitrate reduction were the distinctive features of these waters. The whole-genome sequencing of one of the bacterial isolates which dominated among culturable organisms revealed the presence of a novel subspecies of *B. paralicheniformis*, differing from the relative type strain given the DNA sequence, genome organization, and more diverse physiological traits being essential for survival under life-limiting conditions.

## MATERIALS AND METHODS

### Thermal brine samples.

**FIG 7:**
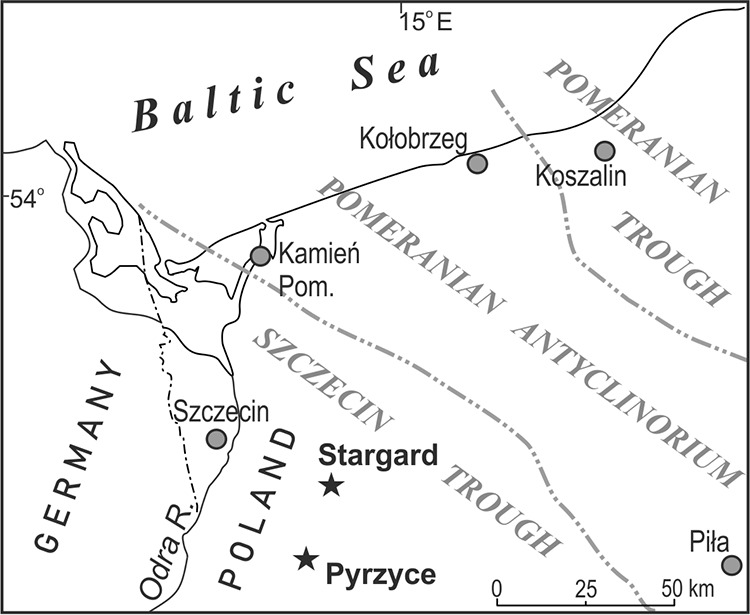
Outlook of the sampling sites.

**FIG 8:**
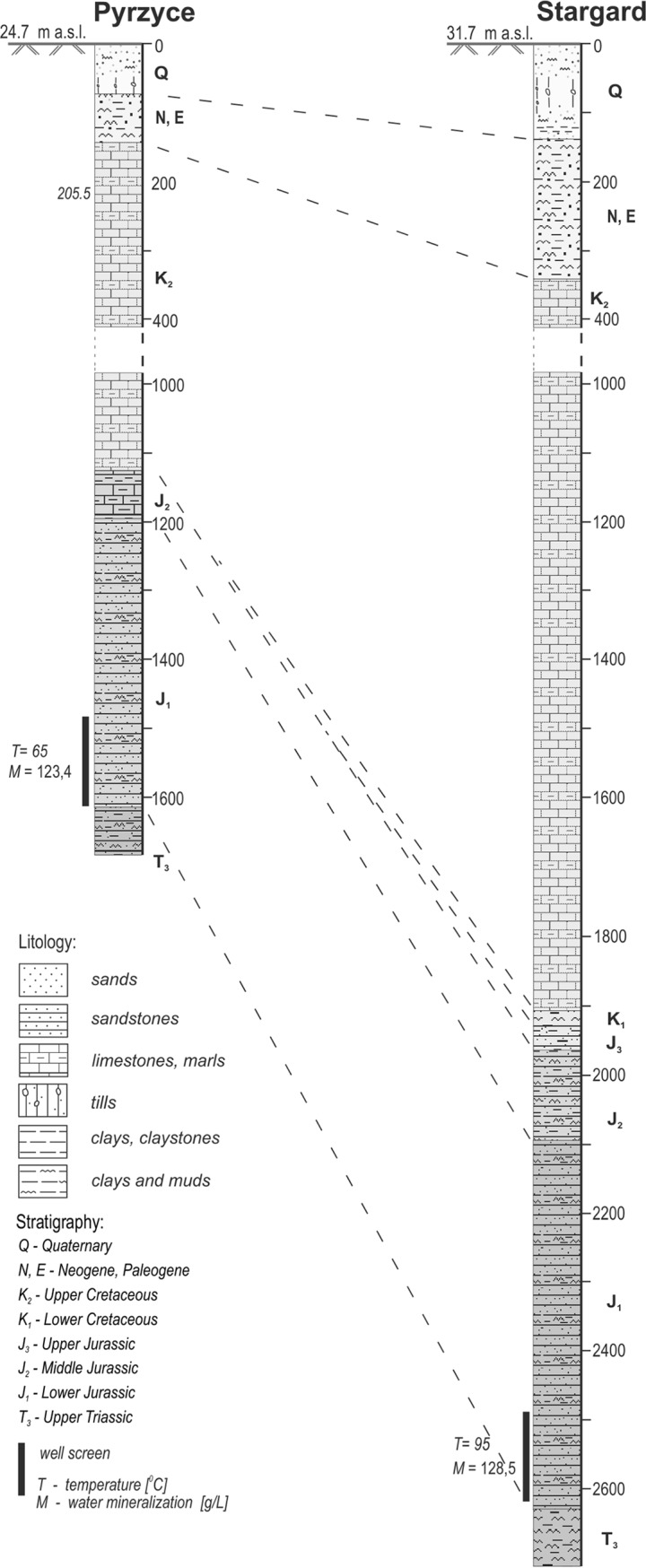
Geological columns of Pyrzyce GT-1 and Stargard GT-2.

Samples were taken in October 2016 from two production wells (three subsamples for each borehole) located at the geothermal heat plants in Pyrzyce (GT-1, drilling date 1992, sample codes P1 to P3, coordinates 53.149444 N, 14.909166 E) and Stargard (GT-2, drilling date 2003, sample codes S1 to S3, coordinates 53.349588 N, 15.0036161 E) being not less than 30 km apart (Fig. 7). Both production wells were situated within the Szczecin Trough above 25 km from the nearest current or historical mine, which enabled sampling of the minimally impacted strata. The mineral waters were extracted from the lower Jurassic sandstones from a depth of 1,640 mbgs in Pyrzyce and 2,578 mbgs in Stargard (Fig. 8). The borehole in Pyrzyce has operated continuously since its start in 1997. The borehole located in Stargard experienced some technical problems which resulted in a break of a few months during the year 2010. Since then, it has worked continuously, which excludes the possibility of aquifer contamination caused by humans.

Samples were taken from the head of the borehole. The valve outlet was flame sterilized. Sample containers were filled with brine, avoiding air bubbles, and tightly sealed. Brines used for anaerobic culturing and DNA extraction were collected in sterile borosilicate glass bottles with a reducing agent, l-cysteine-HCl (0.8 mM final concentration). Samples to be analyzed for ionic composition, as well as for total inorganic/total organic carbon (TIC/TOC) ratios, were collected in glass bottles. Nitric acid was used to preserve samples for cation analyses (1.5 mM final concentration). For stable isotope analyses, samples were collected in polypropylene bottles. All samples were transported at 4°C. The analyses started within 12 h after collection.

### Chemical and physical analyses of brine samples.

Total dissolved solids were measured by the gravimetric method. Concentrations of inorganic compounds (cations, anions, and H_2_S) were determined according to Polish norms of water quality testing by the following methods: spectrophotometric (for H_2_S) using spectrophotometer SQ118 (Merck, Darmstadt, Germany), titration (for HCO_3_^−^ and Mg^2+^), flame photometry (for Na^+^ and Ca^2+^) using the flame photometer BWB XP (BWB Technologies Ltd., Essex, England), and ion-exchange chromatography (for Cl^−^ and SO_4_^2−^) using the Dionex ICS 3000 (Dionex, Sunnyvale, CA, USA). Total organic carbon (TOC) was measured with the TOC 5000 analyzer (Shimadzu, Kyoto, Japan). TOC values were calculated by subtracting inorganic carbon concentration (IC) from total carbon concentration (TC). Organic nitrogen was calculated by subtracting ammonia nitrogen from total Kjeldahl nitrogen (TKN), which was determined in the Turbotherm system (Gerhardt, Königswinter, Germany). Ammonia nitrogen concentration was measured by the Nessler method using the Spectroquant SQ 118 (Merck, Darmstadt, Germany) after distillation in the Vapodest 20 (Gerhardt, Königswinter, Germany). Nitrate concentration was determined using the Spectroquant SQ 118 after extracting nitrate from the sample with 2.0 M KCl. Total phosphorous (TP) was measured after mineralization, using molybdenum blue method by UV-visible (UV-Vis) spectrophotometry. All analyses were performed in triplicates; the mean values were calculated. Data regarding pH and temperature in the aquifer’s sediments were measured by others (65–67) according to PN-ISO 5667-11, 2004 (68).

To determine the age of water, the isotope ratios (^18^O/^16^O and ^2^H/^1^H) were measured by dual-inlet isotope ratio mass spectrometry with the gas chromatography-combustion-mass spectrometry (GC-C-MS) instrument (Finnigan Mat, San Jose, CA, USA) and were reported as the relative deviations from the VSMOW standard. Tritium concentration measurement was conducted in order to assess if the studied water contained an admixture of waters originating from infiltration in the “post-bomb” era (i.e., since 1650). Tritium concentration was measured by electrolytic enrichment followed by liquid scintillation spectrometry in the TRI-CARB liquid scintillation counter (PerkinElmer, Waltham, MA, USA). Tritium concentrations were reported as tritium units (TU), where one TU corresponded to a ^3^H/^1^H ratio of 10^−18^. The uncertainties for the stable isotopes were 0.01‰ and 1‰, respectively. Both analyses were performed in the laboratory of the Environmental Physics Group at the AGH University of Science and Technology in Kraków, Poland.

### Enumeration of microorganisms.

The total number of microbial cells was determined by the acridine orange direct cell counting (AODC) technique. Prior to the analysis, a 10-ml aliquot of each sample (in triplicates) was immediately fixed with formaldehyde (4% final concentration). Microbial cells absorbed on the surface of black polycarbonate filters (MF-Millipore membrane filters, 0.22 μm) were stained with acridine orange (final concentration, 0.005 g/liter) for 3 min. Pieces of filters were immediately placed on slides and viewed at a ×1,000 magnification. Color images were made using digital image processing camera (Nikon DS-Fi1) and viewed using the software package NIS-Elements F v. 3.0. The enumeration of cells was conducted manually by counting the cells in at least ten images.

The number of culturable heterotrophic microorganisms was estimated using the pour plate technique. One-milliliter aliquots of each sample were placed aseptically in petri dishes and poured out with medium cooled to a temperature of around 45°C. The composition of medium was as follows: nutrient broth (Biocorp, Warsaw, Poland), 0.1%; agar, 2.5%; and NaCl at a concentration corresponding to the chloride content in a given sample. The content of agar was increased to prevent liquefaction of medium at high temperature. The cultures were incubated for 14 days at 60°C in a GENbox (2.5 liters) under microaerophilic conditions generated by GENbox Microaer sachets (bioMérieux, Marcy-l’Etoile, France). The analysis was performed in triplicates. The number of microorganisms was expressed as CFU per milliliter of sample. Colonies that grew on the plates were chosen randomly for subsequent taxonomic identification. Glycerol stocks of the isolates were prepared and stored at −80°C.

### Extraction of total microbial community DNA.

The total microbial community DNA was isolated as follows: 300 ml of brine from each subsample was filtered through sterile polycarbonate filter membranes (0.22-μm pore size; Millipore). Filters were cut into small pieces, put into Eppendorf tubes, and resuspended in 500 μl lysis buffer (100 mM Tris-HCl [pH 8.0], 100 mM EDTA [pH 8.0], 100 mM phosphate buffer, 1.5 M NaCl, 1% SDS) and supplemented with 20 μg proteinase K. After incubation (1 h, 55°C), DNA was extracted with chloroform (1:1 [vol/vol]). The DNA was precipitated with 0.7 volumes of isopropanol. After centrifugation (10,000 × *g*, room temperature [RT], 30 min), the pellet was washed twice with 70% ethanol, air dried, and dissolved in nuclease-free water. The DNA concentration was measured with a Qubit 2.0 (Invitrogen, Carlsbad, CA, USA) using a Qubit dsDNA HS assay kit (Thermo Fisher Scientific). To exclude DNA contamination linked to materials and reagents, the first negative-control sample was prepared on this stage. Microbial DNA-free water (Qiagen, Hilden, Germany) was filtered and used for DNA extraction (69), the subsequent amplification, and sequencing.

### Next-generation DNA sequencing.

Preparation of bacterial and archaeal 16S rRNA gene amplicon libraries, library quantification, and sequencing as well as subsequent bioinformatic analysis were performed as previously described (51).

Briefly, the amplicons were prepared using bacterial (357F, CCT ACG GGA GGC AGC AG; 786R, ACC AGG GTA TCT AAW C [20, 70]) and archaeal (513F, GGT GYC AGC CGC CGC GGT AA; 915R, GTG CTC CCC CGC CAA TTY CT [51]) primers. At the stage of library preparation, the second negative control was prepared in which microbial DNA-free water (Qiagen, Hilden, Germany) was used instead of environmental DNA (69). Separate bacterial and archaeal libraries were created by mixing equal quantities of 16S rRNA gene amplicons and additional libraries containing amplicons obtained from the first and the second negative controls. Libraries were purified using Agencourt AMPure XP (Beckman Coulter, Brea, CA, USA) and evaluated on an Agilent 2100 Bioanalyzer with a high-sensitivity DNA analysis kit (Agilent Technologies, Waldbronn, Germany). Sequencing was performed on a MiSeq instrument (Illumina, San Diego, CA, USA) using MiSeq reagent kit v3 (600 cycles).

After a quality trimming of FastQ files and correction of reads, the paired-end reads were generated, and the contigs were analyzed with mothur v 1.39 (71) as recommended by the MiSeq SOP page (http://www.mothur.org/wiki/MiSeq_SOP). Sequences obtained with the bacterial and the archaeal specific primers were assorted based on the alignment using SILVA v.132. Putative chimeric sequences, identified with UCHIME and Perseus, were removed. Operational taxonomic units (OTUs) were constructed via the unweighted pair group method with arithmetic mean (UPGMA) at the 0.03 dissimilarity level. Singletons and doubletons were discarded. The remaining OTUs were assigned taxonomic classification, and rarefaction curves for the obtained OTUs were calculated. Reads from negative controls were removed from the data set.

Richness estimator (OTUs observed) and diversity index (Shannon-Wiener) were calculated at the 0.03 dissimilarity level (72) with the mothur software for 1,000-fold randomized subsamples of 1,400 sequences per sample for *Bacteria*. This step was skipped for *Archaea*, as only one archaeal OTU was obtained.

To test differences in the community compositions in samples from different wells (Pyrzyce versus Stargard), a nonparametric multivariate analysis of variance (PERMANOVA) was applied using vegan in R (73).

Phylogenetic trees of the most abundant bacterial and archaeal OTUs based on the 16S rRNA gene were constructed using MEGA ver. 5.2 and the maximum likelihood method with the general time reversible model and were based on the 426 bacterial and 386 archaeal nucleotide positions.

### Molecular identification of bacterial isolates.

Bacteria were grown in a liquid medium under microaerophilic conditions as described above. Cells were harvested after 24 h of growth at 60°C from 5 ml of a bacterial suspension by centrifugation for 10 min at 5,000 × *g*. Total genomic DNA was extracted from the cells using the DNeasy blood and tissue kit with a modified protocol for DNA extraction from Gram-positive bacteria (Qiagen, Hilden, Germany). The 16S rRNA gene was amplified by PCR in a reaction mixture containing 1 U Dream *Taq* green polymerase (Fermentas, St. Leon-Rot, Germany), 0.2 mM deoxynucleoside triphosphate (dNTP) mixture, 1× Dream *Taq* green buffer, 1.5 mM MgCl_2_, 0.25 μM of each universal primer, i.e., 27F (5′-AGA GTT TGA TCM TGG CTC AG-3′) (74) and 1492R (5′-TAC GGY TAC CTT GTT ACG ACT T-3′) (75), and 1 μl of genomic DNA (total volume of 20 μl). The initial denaturation performed at 95°C for 3 min was followed by 30 amplification cycles (denaturation at 95°C for 30 s, annealing at 52°C for 20 s, and extension at 72°C for 1 min 40 s) and a final extension at 72°C for 5 min. PCR amplicons were inspected on an agarose gel (1% [wt/vol]) stained with Midori green DNA stain (Nippon Genetics Europe GmbH, Düren, Germany). PCR products were sequenced with primer 27F, using the BigDye Terminator v 3.1 cycle sequencing kit (Applied Biosystems, Thermo Fisher Scientific, Waltham, MA, USA) in accordance with the manufacturer’s procedure. Capillary electrophoresis was performed by the Oligo sequencing laboratory (IBB PAS, Warsaw, Poland). The taxonomic affiliation of the strains was determined using the EzBioCloud online tool (76) with a 98.7% cutoff (77).

### Physiological properties of *Bacillus* strain TS6.

Growth of TS6 was tested at the temperature from 10 to 60°C, steps of 10°C, using 0.1% nutrient broth. The pH range was investigated from pH 3.0 to 11.0 in steps of 1.0 pH using the same medium buffered with 0.1 M citrate-phosphate buffer (pH 3 to 7), Tris (pH 8), and NaCO_3_ (pH 9, 10, and 11). The pH measurements were taken at a temperature of 50°C. NaCl tolerance was determined using 0.1% nutrient broth supplemented with 0% to 10% NaCl (wt/vol) in steps of 1%. Urease production was tested using Stuart’s medium containing (g/liter) urea, 20; Na_2_HPO_4_, 9.5; KH_2_PO_4_, 9.1; yeast extract, 0.1; and phenol red, 0.01. Carbon source utilization was assessed using the OmniLog Data Collection system (Biolog). Strain TS6 was cultured overnight on Biolog universal growth plates and prepared according to the manufacturer’s instructions for the GEN III MicroPlate test panel and for phenotype array plates PM1 and PM2a (Biolog). An active growing culture of TS6 was sent for the cellular fatty acid analysis at DSMZ, Braunschweig, Germany. Fatty acid methyl esters were obtained from 40 mg cells scraped from petri dishes by saponification, methylation, and extraction (78, 79). The fatty acid methyl ester mixtures were separated on a gas chromatograph (Agilent 6890N; Hewlett-Packard Co., Palo Alto, CA, USA) and identified using the Sherlock microbial identification system (MIS) (MIDI; Microbial ID, Newark, DE, USA). Peaks were automatically integrated, and fatty acid names and percentages were calculated by the MIS standard software (Microbial ID). To assess the thermotype of the investigated strain, the heat adaptation index (HAI) and anteiso- to iso-C_15:0_ FA ratio (a15/i15) were calculated (62, 80).

### *Bacillus* strain TS6 genome sequencing and analysis.

DNA extracted from the strain TS6, representing the most frequent isolates retrieved, was sent for whole-genome sequencing at the University of Birmingham, Great Britain. Genomic DNA libraries were prepared using a Nextera XT Library Prep kit (Illumina, San Diego, CA, USA) according to the manufacturer’s protocol with the following modifications: 2 ng of DNA instead of 1 ng was used as input, and PCR elongation time was increased to 1 min from 30 s. DNA quantification and library preparation were carried out on a Microlab STAR automated liquid handling system (Hamilton, Bonaduz, Switzerland). Pooled libraries were quantified using the Kapa library quantification kit for Illumina (Kapa Biosystems, Wilmington, MA, USA) on a LightCycler 96 quantitative PCR (qPCR) machine (Roche, Basel, Switzerland).

Libraries were sequenced on the Illumina HiSeq system using a 250-bp paired-end protocol. Reads were adapter trimmed using Trimmomatic 0.30 with a sliding window quality cutoff of Q15 (81). *De novo* assembly was performed on samples using SPAdes version 3.9.1 (82); assembly metrics were calculated by Quast (83) and contigs were annotated using Prokka 1.11 (84). Further annotation was made using the NCBI Prokaryotic Genome Annotation Pipeline (PGAP).

Average nucleotide identity (ANI) calculator, available from EzBioCloud (85), was used to identify the most similar genome among the seven complete genomes of *B. paralicheniformis* available at NCBI. Then, the TS6 genome was compared to the reference genome of *B. paralicheniformis* 14DA11 and to its closest type strain, *B. paralicheniformis* KJ-16, using the genome-to-genome distance calculator v 2.0 and formula 2 (86) at DSMZ (https://ggdc.dsmz.de).

Multiple-genome alignment was performed in Mauve (Multiple Genome Alignment, v 20150226) according to Edwards and Holt (87). For this purpose, genomic alignments were performed for the two draft genomes (TS6 and KJ-16) against a complete reference genome sequence of *B. paralicheniformis* 14DA11.

To get insight into the functional features encoded in the genomes of *B. paralicheniformis* TS6 and *B. paralicheniformis* KJ-16, amino acid sequences predicted by Prokka were used as input to GenBank Trans Extractor (http://www.bioinformatics.org/sms2/genbank_trans.html) to obtain predicted protein translations of the DNA sequence. These sequences were assigned KEGG orthology (KO) identifiers via “annotate sequence” (https://www.kegg.jp/kegg/tool/annotate_sequence.html) and were used for the subsequent reconstruct pathway analysis (https://www.kegg.jp/kegg/tool/map_pathway.html).

### Data availability.

Sequencing data were deposited in the Sequence Read Archive at NCBI under BioProject accession PRJNA476244. The 16S rRNA gene fragments of the bacterial strains obtained in this study were deposited to GenBank under the accession numbers MK495712 to MK495721. This whole-genome shotgun project has been deposited at DDBJ/ENA/GenBank under the accession number SHMY00000000. The version described in this paper is version SHMY01000000.
